# Combined fetal inflammation and postnatal hypoxia causes myelin deficits and autism‐like behavior in a rat model of diffuse white matter injury

**DOI:** 10.1002/glia.23216

**Published:** 2017-09-19

**Authors:** Erik van Tilborg, E. J. Marijke Achterberg, Caren M. van Kammen, Annette van der Toorn, Floris Groenendaal, Rick M. Dijkhuizen, Cobi J. Heijnen, Louk J. M. J. Vanderschuren, Manon N. J. L. Benders, Cora H. A. Nijboer

**Affiliations:** ^1^ Laboratory of Neuroimmunology and Developmental Origins of Disease University Medical Center Utrecht Utrecht 3584EA The Netherlands; ^2^ Department of Animals in Science and Society Division of Behavioural Neuroscience, Faculty of Veterinary Medicine, Utrecht University Utrecht 3584CM The Netherlands; ^3^ Biomedical MR Imaging and Spectroscopy Group Center for Image Sciences, University Medical Center Utrecht 3584 CJ The Netherlands; ^4^ Department of Neonatology University Medical Center Utrecht Utrecht 3584EA The Netherlands; ^5^ Laboratory of Neuroimmunology Department of Symptom Research, University of Texas MD Anderson Cancer Center Houston Texas 77030

**Keywords:** astrocytes, autism‐like behavior, microglia, oligodendrocytes, preterm birth

## Abstract

Diffuse white matter injury (WMI) is a serious problem in extremely preterm infants, and is associated with adverse neurodevelopmental outcome, including cognitive impairments and an increased risk of autism‐spectrum disorders. Important risk factors include fetal or perinatal inflammatory insults and fluctuating cerebral oxygenation. However, the exact mechanisms underlying diffuse WMI are not fully understood and no treatment options are currently available. The use of clinically relevant animal models is crucial to advance knowledge on the pathophysiology of diffuse WMI, allowing the definition of novel therapeutic targets. In the present study, we developed a multiple‐hit animal model of diffuse WMI by combining fetal inflammation and postnatal hypoxia in rats. We characterized the effects on white matter development and functional outcome by immunohistochemistry, MRI and behavioral paradigms. Combined fetal inflammation and postnatal hypoxia resulted in delayed cortical myelination, microglia activation and astrogliosis at P18, together with long‐term changes in oligodendrocyte maturation as observed in 10 week old animals. Furthermore, rats with WMI showed impaired motor performance, increased anxiety and signs of autism‐like behavior, i.e. reduced social play behavior and increased repetitive grooming. In conclusion, the combination of fetal inflammation and postnatal hypoxia in rats induces a pattern of brain injury and functional impairments that closely resembles the clinical situation of diffuse WMI. This animal model provides the opportunity to elucidate pathophysiological mechanisms underlying WMI, and can be used to develop novel treatment options for diffuse WMI in preterm infants.

## 1 INTRODUCTION

White matter injury (WMI) is a major complication in infants born prematurely. Diffuse, noncystic WMI as characterized by decreased white matter volumes and low fractional diffusion anisotropy (FA) values detected by MRI, affects the majority of WMI patients in neonatal intensive care units (Back & Miller, 2014). Studies on postmortem brain tissue of preterm infants indicated that myelination deficits in diffuse WMI are mediated by impaired differentiation of immature oligodendrocytes, without specific loss of oligodendrocytes (Billiards et al., 2008; Buser et al., 2012; Verney et al., 2012). In contrast, several other studies found evidence of oligodendrocyte‐specific apoptosis in the brains of preterm infants (Haynes et al., 2003; Robinson, Li, Dechant, & Cohen, 2006). Additionally, activation of microglia and astrogliosis are important pathophysiological hallmarks of diffuse WMI (Buser et al., 2012; Haynes et al., 2003; Verney et al., 2012). Functionally, WMI is associated with behavioral, cognitive, motor and psychological problems, like autism spectrum disorders (ASD), later in life (Back & Miller, 2014; Dudova et al., 2014; Guo et al., 2017; Johnson, Hollis, Kochhar, Hennessy, Wolke, & Marlow, 2010; Joseph et al., 2017; Kuzniewicz et al., 2014; Limperopoulos et al., 2008; Peyton et al., 2016, 2017; Pritchard et al., 2016; Treyvaud et al., 2013). While antenatal administration of magnesium sulfate in women at risk for preterm delivery reduces the risk of cerebral palsy in the fetus (Crowther, Hiller, Doyle, & Haslam, 2003; Doyle, Crowther, Middleton, Marret, & Rouse, 2009; Gano et al., 2016; Rouse et al., 2008), limited treatment options are currently available to protect preterm infants against diffuse WMI. Moreover, the mechanisms underlying diffuse WMI are not completely understood (van Tilborg et al., 2016). Therefore, it is crucial that clinically relevant animal models are being developed to allow the investigation of new therapies.

Important risk factors for diffuse WMI include fetal or perinatal inflammatory insults and postnatal hypoxic/hyperoxic events (Chau, Brant, Poskitt, Tam, Synnes, & Miller, 2012; Procianoy & Silveira, 2012; Resch et al., 2012; Shah et al., 2008; Shankaran, Langer, Kazzi, Laptook, & Walsh, 2006; Tsuji et al., 2000). It has been proposed that exposure to multiple perinatal hits plays a crucial role in the etiology of WMI, with a first insult sensitizing the developing brain to subsequent insults that aggravate injury (Kaindl, Favrais, & Gressens, 2009; Van Steenwinckel et al., 2014). This notion is supported by clinical data indicating that exposure to multiple insults dramatically increases the risk of white matter abnormalities (Korzeniewski et al., 2014; Leviton et al., 2013). In the present study, we investigated the pathology and outcome in a novel rat model of diffuse WMI in preterm infants, in which two clinically relevant perinatal hits, i.e. fetal inflammation and postnatal hypoxia, are combined during relevant stages of brain development. Neuropathology was studied by histology and MRI. Furthermore, functional outcome on several relevant modalities was investigated by behavioral paradigms for motor coordination, cognitive functioning, anxiety‐like behavior and autism‐like behavior.

## 2 MATERIALS AND METHODS

### 2.1 Animals

All procedures were performed according to Dutch (“Wet op de dierproeven”, 1996) and European regulations (Guideline 86/609/EEC) and were approved by the Animal Ethics Committee of Utrecht University.

Wistar rats (Envigo, Horst, The Netherlands) were kept under standard housing conditions. Timed‐pregnant animals received intraperitoneal injections of 100 μg/kg lipopolysaccharide (LPS) (from E. Coli O55:B5, L2880, Sigma, St. Louis, MO) in 1.0 ml/kg saline or saline (vehicle) on E18 and E19. Maternal LPS injections have previously been shown to induce cytokine expression in the fetal brain (Cai, Pan, Pang, Evans, & Rhodes, 2000). Increasing the dosage of LPS (up to 500 μg/kg) resulted in high rates of stillbirth and/or high perinatal mortality in the offspring (data not shown). At P4, offspring was randomly placed in a temperature‐controlled hypoxic chamber containing 8% O_2_ in N_2_ or in a normoxic temperature‐controlled environment (i.e., not in the homecage to circumvent potential confounding effects of maternal deprivation) for 140 min. Pups of both sexes were used. Specific animal numbers are mentioned in the figure captions.

### 2.2 Immunohistochemistry and analysis

At P5, P18, P30 (post‐MRI see below) and P69, animals received an overdose of 300 mg/kg pentobarbital and were transcardially perfused with phosphate buffered saline (PBS), followed by 4% paraformaldehyde (PFA) in PBS. Brains were fixed in 4% PFA for 24 hr and embedded in paraffin. 8μm coronal sections were cut, deparaffinized and rehydrated prior to antigen retrieval by heating sections to 95°C in sodiumcitrate buffer (0.01M, pH 6) for 9 min. Sections were blocked with 5% normal serum or 2% bovine serum albumin (BSA) and 0.1% saponin in PBS and incubated overnight with primary antibodies diluted in PBS (mouse‐anti‐CNPase, 1:500, Abcam; mouse‐anti‐GFAP, 1:250, Cymbus; rabbit‐anti‐Iba1, 1:500, WAKO; rabbit‐anti‐Ki67, 1:300, Abcam; mouse‐anti‐MBP, 1:1000, Sternberger Monoclonals; rabbit‐anti‐NF200, 1:400, Sigma; rabbit‐anti‐Olig2, 1:500, Chemicon; mouse‐anti‐Olig2, 1:500, Millipore). Sections were then incubated with alexafluor‐594 or −488 conjugated secondary antibodies (Life Technologies, Carlsbad, CA) for 1 hr at room temperature, followed by DAPI counterstaining.

For 3,3′‐diaminobenzidine (DAB) staining, sections were deparaffinized, incubated in 3% H_2_O_2_ in methanol, and sections were blocked in 20% normal horse serum and 0.5% TritonX in PBS. After overnight incubation with mouse‐anti‐MBP primary antibody (see above, 1:1600), sections were incubated with biotinylated horse‐anti‐mouse secondary antibody (Vector Laboratories, Peterborough, UK) for 45 min at room temperature, and revealed by Vectastain ABC kit (Vector Laboratories) and DAB.

For cell death measurements, a terminal deoxynucleotidyl transferase dUTP nick end labeling (TUNEL) cell death detection kit (Roche, Basel, Switzerland) was used on P5 brain sections according to manufacturer's instructions. Prior to the TUNEL assay, sections were immunofluorescently stained for Olig2, as described above.

For each animal, micrographs from both hemispheres were obtained in a blinded fashion using a Cell Observer microscope (Zeiss, Oberkochen, Germany). For MBP stainings and CNPase/Olig2 stainings, in each hemisphere a 20X picture was taken at a fixed distance from the external capsule in the barrel field of the sensory cortex. For TUNEL/Olig2 and Ki67/Olig2 stainings, two 20X pictures of the corpus callosum were acquired in each hemisphere. Similarly, for Iba1 stainings in each hemisphere two 20X pictures were taken in the corpus callosum and in the motor cortex. For GFAP stainings, a 10X picture of the cingulum and a 20X picture just medial to the cingulum were acquired in each hemisphere. The MBP‐DAB staining was photographed on an AxioLab microscope (Zeiss, Oberkochen, Germany). Of each hemisphere, a 10X picture was acquired of the external capsule and sensory cortex and a 40X photograph was acquired at a fixed distance from the external capsule.

Densitometry (controlled for background signal) and threshold analyses were performed using ImageJ software v1.47. Segmentation and structural analysis of MBP stainings were performed by ImageJ plugin DiameterJ (Hotaling, Bharti, Kriel, & Simon, 2015). For each animal, values of all acquired pictures were averaged. Coherency of NF200^+^ axons was measured using the OrientationJ plugin for ImageJ (Fonck et al., 2009). Cell counting was performed manually using ImageJ or Axiovision (Zeiss, Oberkochen, Germany) software and controlled for measured area. Criteria for cell inclusion were clear presence of a DAPI^+^ nucleus, together with clear Ki67^+^/Olig2^+^, CNPase^+^/Olig2^+^, CNPase^‐^/Olig2^+^, or Iba1^+^ staining. The particle analysis function of ImageJ software was used to assess different morphological aspects of microglia, as described earlier (Zanier, Fumagalli, Perego, Pischiutta, & De Simoni, 2015). Manually, correctly identified microglia were selected and shape descriptor parameters were measured. For each animal, the values of all measured microglia were averaged.

### 2.3 Quantitative real time reverse transcriptase (RT)‐PCR

At P5, rats were euthanized by an overdose of 300 mg/kg pentobarbital. The cerebrum was flash frozen in liquid nitrogen and pulverized. Total RNA was isolated using TRIzol (Invitrogen, Paisley, UK). cDNA was synthesized with SuperScript Reverse Transcriptase (Invitrogen). The PCR reaction was performed with iQ5 Real‐Time PCR Detection System (Bio‐Rad) using primers for PDGF‐A (forward: CCCATGTGTGGAGGTGAAG; reverse: TGGCTTCTTCCTGACATACTCC), PDGF‐B (forward: ATCCGCTCCTTTGATGACCT; reverse: TCAGCCCCATCTTCGTCTAC), BMP4 (forward: TGAGGAGTTTCCATCACGAA; reverse: CACCTGCTCCCGAAATAGC), Jagged1 (forward: CTGCGTGGTCAATGGAGACT; reverse: CAAAACCAGGGGCACATTCG), GLT‐1 (forward: GAGCATTGGTGCAGCCAGTATT; reverse: GTTCTCATTCTATCCAGCAGCCAG; Hammad, Althobaiti, Das, & Sari, 2017) and GLAST (forward: AATGAAGCCATCATGAGATTGGT; reverse: CCCTGCGATCAAGAAGAGGAT; Zschocke et al., 2005). Data were normalized to the relative expression of β‐actin (forward: CACTATCGGCAATGAGCGGTTCC; reverse: CAGCACTGTGTTGGCATAGAGGTC).

### 2.4 Postmortem MRI

At P30, animals received transcardial perfusion with PBS, followed by 4% PFA in PBS. The head of the animal was removed and postfixed for 3 days in 4% PFA, followed by incubation in PBS with 0.05% sodium azide for a minimum of 10 days. Prior to scanning, all skin and muscle tissue was removed from the skull. Brains were scanned overnight in a 9.4T MR system (Varian, Palo Alto, CA, USA) to obtain anatomical images at 100 μm isotropic resolution using a balanced steady state free precession (BSSFP) sequence (repetition time (TR)/echo time (TE) 15.4/7.7 ms, flip angle 40°, 320 × 160 × 190 matrix, field‐of‐view (FOV) 32 × 16 × 19 mm^3^, 6 averages, pulse angle shift 0°, 90°, 180°, and 270°), and diffusion parameter maps at 150 μm isotropic resolution using diffusion tensor imaging (DTI) with *b* = 3,842 s/mm^2^ (TR/TE 500/32.4 ms, 220 × 128 × 108 matrix, FOV 33 × 19.2 × 16 mm^3^, Δ/δ 15/4 ms, 60 diffusion‐weighted images in noncollinear directions and 5 images without diffusion weighting (*b* = 0), number of averages 1, total number of images 65). Data were analyzed using the FMRIB (Oxford Center for Functional MRI of the Brain) Software Library (FSL) (Jenkinson, Beckmann, Behrens, Woolrich, & Smith, 2012) and Matlab software. Following registration to an anatomical rat brain template, we measured mean diffusivity and FA in atlas‐based regions of interest (ROIs) (Supporting Information Figure S1). The size of the ROIs was calculated from the anatomical images and are expressed relative to the cerebrum size. Relative white matter volumes were calculated by setting a threshold on BSSFP scans: signal intensities between 0.91 and 1.24 were considered white matter. After MRI, brains were used for further immunohistochemical analyses as described above.

### 2.5 Behavioral assessment

All behavioral experiments and analyses were performed by experienced researchers blinded to experimental conditions. In between testing trials, testing apparatuses were thoroughly cleaned. Except for the rotarod task, all behavioral tests were performed under red light conditions.

#### 2.5.1 Rotarod

Gross motor skills and coordination were determined using the rotarod (RTR) task . At the age of 5 weeks, rats were trained on two consecutive days to remain on a rotating rod (5 rotations per minute (rpm)) for 150 s. On days 3 and 4, animals were placed on the RTR, with the rotation speed increasing from 5 to 40 rpm over the course of a 300 second trial. The time spent on the rod (averaged over the two test trials) was recorded, which is considered a measure of motor performance.

#### 2.5.2 Novel object recognition task

Memory function was assessed using the novel object recognition task (NORT) at an age of 9 weeks . Animals were placed in a test cage without bedding containing two identical objects (white/transparent spherical glass globe or upside‐down dark‐gray ceramic bowl) for 10 min, after which the animal was placed back into its homecage. One hour later, animals were placed back in the test cage for 5 min with one object being replaced with a novel object. Exploration behavior was videotaped and the time that the animal spent exploring the familiar and novel objects was recorded. Object types (globe/bowl) on a randomly assigned side (left/right) of the test cage were randomly designated as familiar/novel objects, to exclude a potential bias of side‐ or object preference. Data are presented as percentage of the time spent exploring the novel object relative to the total exploration time on both objects.

#### 2.5.3 Delayed spontaneous alteration (T‐maze)

Working memory was assessed at the age of 6 weeks by measuring delayed spontaneous alteration in a T‐maze paradigm . Each trial in this test consisted of two runs in a T‐shaped maze: a sample run and a choice run. During the sample run, the animal was placed in the starting arm of the T‐maze. The investigator waited until the animal entered one of either goal arms (max. 2 min) and closed the arm, forcing the animal to remain in the same goal arm for 30 s. Immediately after the sample run, the animal was placed back into the starting arm for the choice run and it was recorded whether the animal first started exploring the previously explored arm or the unexplored arm. If the animal started exploring the unexplored arm first, this was recorded as correct alteration. For 4 consecutive days, animals performed 2 trials per day. Between each run, feces and urine were removed from the maze. Preference for the alternating arm is expressed as percentage of the total number of choice runs choosing the alternate arm, relative to the previously explored arm.

#### 2.5.4 Repetitive grooming

Repetitive grooming behavior was assessed at the age of 5 weeks by placing the rats in a transparent cage containing only fresh bedding material for 20 min. The animals were videotaped and time spent grooming was recorded .

#### 2.5.5 Anxiety‐related behavior

We used two paradigms to investigate anxiety‐related behavior at the age of 7 weeks. In the open field test, animals were placed in a circular arena with a diameter of 1 m for 5 min and locomotion behavior was automatically tracked using Ethovision software (Noldus, Wageningen, The Netherlands). The arena was divided into two sections: the outer rim of the arena (12.5 cm) and the inner zone (diameter of 75 cm). The number of times that animals left the outer zone (sheltered by the walls of the arena) and entered the inner zone was recorded. This is considered a measure of nonanxious behavior.

For the elevated plus maze (EPM), animals were placed in the middle of a plus‐shaped maze with a 10 cm × 10 cm center (light intensity: 8–10 lux) connecting two opposite open arms (length: 50 cm; 16 lux) and two opposite arms closed with 30 cm high walls (length: 50 cm; 0 lux), 1 m above a dimly lit floor for five minutes. Animal movement was tracked using Ethovision software (Noldus, Wageningen, The Netherlands). The time spent in the open arms is considered a measure of nonanxious behavior.

#### 2.5.6 Social play behavior

Impaired social play behavior is associated with ASD (Jordan, 2003). At the age of 4 weeks, social play behavior was assessed as previously described (Achterberg, van Kerkhof, Damsteegt, Trezza, & Vanderschuren, 2015). Briefly, animals were habituated to the 40 cm × 40 cm test chamber for 10 min on 2 consecutive days. On the third day, animals were socially isolated for 2.5 hr before placing them into the test chamber together with a nonfamiliar, weight‐ and sex‐matched animal of the same experimental group. For 15 min, social play behavior was videotaped and later scored for pouncing (attempting to nose or rub the nape of the other animals neck), pinning (standing over the other animal while the other is lying on the floor with its dorsal surface) and general social exploration (e.g., sniffing) using Observer software (Noldus, Wageningen, The Netherlands).

### 2.6 Statistics

Statistics were performed using Graphpad Prism v6.02. For all parameters within this study, the presence of sex‐specific effects were first investigated by two‐way ANOVA with sex and experimental group as dependent variables. In case of a significant effect of sex or an interaction between experimental group and sex, data were further analyzed by multiple comparisons with Bonferroni correction and data are presented for males and females separately. In case there was no significant effect of sex, males and females were pooled. To compare control vs. WMI groups, independent samples *t*‐tests were used and in case of unequal variances Welch's corrections were applied. When comparing more than 2 groups, one‐way ANOVA was performed, followed by post‐hoc multiple comparisons with Bonferroni correction. Correlations were calculated by linear regression. Overall, *p* < .05 was considered statistically significant and data are presented as mean with *SEM*.

## 3 RESULTS

### 3.1 The combination of fetal inflammation and postnatal hypoxia causes delayed myelination in rats

**Figure 1:**
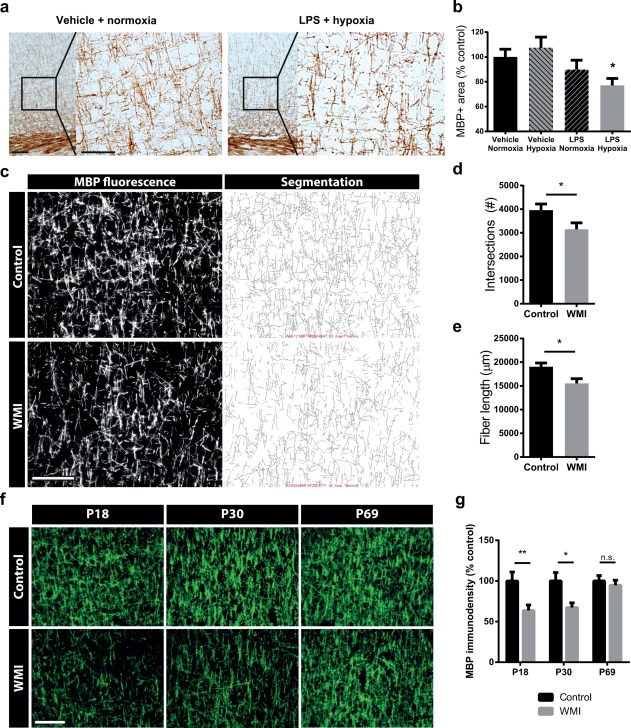
The combination of fetal LPS exposure and postnatal hypoxia causes delayed myelination in neonatal rats. (a) Representative images of MBP (DAB) stainings of animals from mothers injected with vehicle that were exposed to postnatal normoxia at P4 or animals from mothers injected with LPS (fetal inflammation) that were exposed to postnatal hypoxia at P4. Scale bars: 100 μm (left), 50 μm (inset). (b) Quantification of MBP^+^ area in animals from mothers injected with vehicle that were expose to postnatal normoxia (*n* = 9) or to postnatal hypoxia at P4 (*n* = 7), and animals from mothers injected with LPS (fetal inflammation) that were exposed to postnatal normoxia (*n* = 8) or postnatal hypoxia (*n* = 16) at P4. (c) Fluorescent stainings for MBP (left panels) and automated segmentation (right panels) of the sensory cortex in P18 control and WMI animals. Scale bar: 100 μm. (d‐e) Quantification of intersections between myelinated axons (d) and fiber length (e) to assess the complexity of cortical myelination. These measures were calculated from automated segmentation images from MBP fluorescent stainings in the sensory cortex of P18 rats. Animal numbers: control *n* = 10; WMI *n* = 16. (f) Fluorescent stainings for MBP in the cortex of control and WMI animals at P18, P30, and P69 reveal delayed myelination. Scale bar: 100 μm. (g) MBP immunodensity of control vs. WMI animals at P18 (*n* = 10; *n* = 17), P30 (*n* = 6; *n* = 6), and P69 (*n* = 10; *n* = 10), normalized to control values. *: *p* < .05; **: *p* < .01; n.s.: not significant [Color figure can be viewed at wileyonlinelibrary.com]

To assess the effects of fetal inflammation plus postnatal hypoxia on myelination, brain slices were stained for myelin basic protein (MBP). Compared with control animals, we observed no differences in MBP^+^ area in animals that were exposed to only postnatal hypoxia (*p* > .99) or only fetal LPS (*p* > .99) at P18 (Figure 1a,b). However, rat pups exposed to the combination of fetal inflammation and postnatal hypoxia showed a significant decrease in MBP^+^ area in the sensory cortex (*p* = .044), indicating that the combination of both insults is required to induce myelination deficits. Since no myelination deficits were observed in animals exposed to only fetal inflammation or only hypoxia, further experiments were restricted to animals exposed to neither insult or combined fetal inflammation and postnatal hypoxia (from here onwards referred to as control and WMI animals, respectively). A more detailed analysis of cortical myelination was performed using segmentation of MBP images, which revealed a less complex organization of myelinated axons, comprising a reduced number of intersections between myelinated axons (*p* = .042) and reduced fiber lengths (*p* = .020) in WMI animals compared with control animals at P18 (Figure 1c–e). MBP stainings were also performed on P30 and P69 rats to assess the effects of fetal inflammation and postnatal hypoxia on cortical myelination over time. Compared with control animals, WMI rats showed reduced MBP staining in the sensory cortex at the ages of P18 (*p* = .006) and P30 (*p* = .021), which had returned almost to control levels at P69 (*p* = .869) (Figure 1f,g). Collectively, these data demonstrate that the combination of fetal inflammation and postnatal hypoxia induces reduced myelination until past P30.

To study the size of the lateral ventricles and the hippocampus, the thickness of the corpus callosum and microstructural axonal integrity, additional HE, MBP and NF200 stainings were performed on sections of P18 animals. The experimental rat model induced subtle diffuse WMI, as we did not observe differences in gross neuroanatomy as indicated by ventricle size (*p* = .691), hippocampus size (*p* = .499), and corpus callosum thickness (*p* = .871) (Supporting Information Figure S2). Furthermore, WMI animals showed impaired myelination of axons as indicated by a decreased MBP:NF200 ratio (*p* = .016) (Supporting Information Figure S3a,d), whereas we did not observe any signs of axonal damage as assessed by NF200 stainings (*p* = .911) (Supporting Information Figure S3a–c).

### 3.2 Fetal inflammation and postnatal hypoxia induce impaired oligodendrocyte maturation

**Figure 2:**
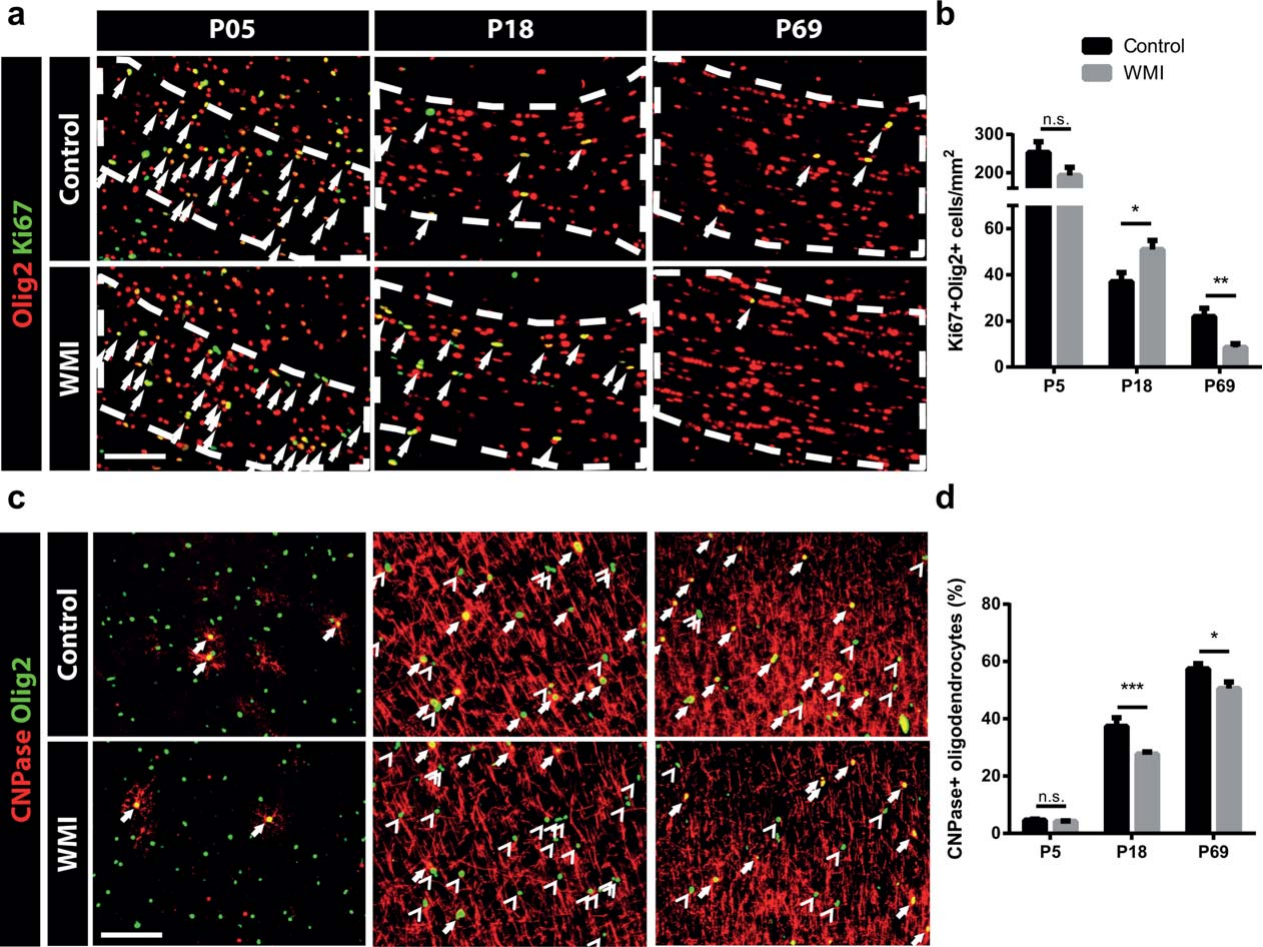
Impaired oligodendrocyte maturation in WMI animals. (a) Corpus callosum (outlined) of control vs. WMI animals stained for oIigodendrocyte marker Olig2 (red) and proliferation marker Ki67 (green). Ki67^+^Olig2^+^ cells indicate immature proliferative oligodendrocytes (arrows). Scale bar: 100 μm. (b) Quantification of Ki67^+^Olig2^+^ cells in the corpus callosum at P5 (control: *n* = 5; WMI: *n* = 6), P18 (control: *n* = 10; WMI: *n* = 17) and P69 (control: *n* = 10; WMI: *n* = 10). (c) Cortical areas of control vs. WMI animals stained for oligodendrocyte differentiation marker CNPase and oligodendrocyte marker Olig2 at P5, P18, and P69. Differentiated CNPase^+^Olig2^+^ oligodendrocytes (arrows) have matured further compared with undifferentiated CNPase^‐^Olig2^+^ oligodendrocytes (arrowheads). Scale bar: 100 μm. (d) Quantification of the percentage of CNPase^+^Olig2^+^ cells in the sensory cortex of control and WMI animals at P5 (*n* = 5; *n* = 6), P18 (*n* = 7; *n* = 17) and P69 (*n* = 10; *n* = 10). *: *p* < .05; **: *p* < .01; ***: *p* < .001; n.s.: not significant [Color figure can be viewed at wileyonlinelibrary.com]

It has been suggested that arrested oligodendrocyte maturation underlies impaired myelination in diffuse WMI in preterm infants (Back & Miller, 2014). To assess oligodendrocyte maturation in animals exposed to combined fetal inflammation and postnatal hypoxia, we performed double‐stainings for developmental stage‐specific markers Ki67 (proliferation marker—oligodendrocytes lose their proliferative capacity during differentiation) or CNPase (more mature oligodendrocytes), together with nuclear oligodendrocyte marker Olig2. Compared with control animals, WMI animals showed no changes in oligodendrocyte maturation at P5 (Ki67: *p* = .117; CNPase: *p* = .259) (Figure 2a–d). Interestingly, at P18 oligodendrocyte maturation was impaired in WMI animals compared with control animals, as illustrated by increased numbers of immature Ki67^+^Olig2^+^ cells in the corpus callosum (*p* = .027) and decreased numbers of CNPase^+^Olig2^+^ cells in cortical areas (*p* < .001) (Figure 2a–d). By P69, the number of Ki67^+^Olig2^+^ cells in the corpus callosum was reduced in WMI animals compared with controls, indicating reduced proliferative capacity of oligodendrocytes after fetal inflammation plus postnatal hypoxia (*p* = .003). Furthermore, the proportion of CNPase^+^ oligodendrocytes in cortical areas remained lower in WMI rats at P69 compared with control animals (*p* = .029) (Figure 2a–d).

**Figure 3:**
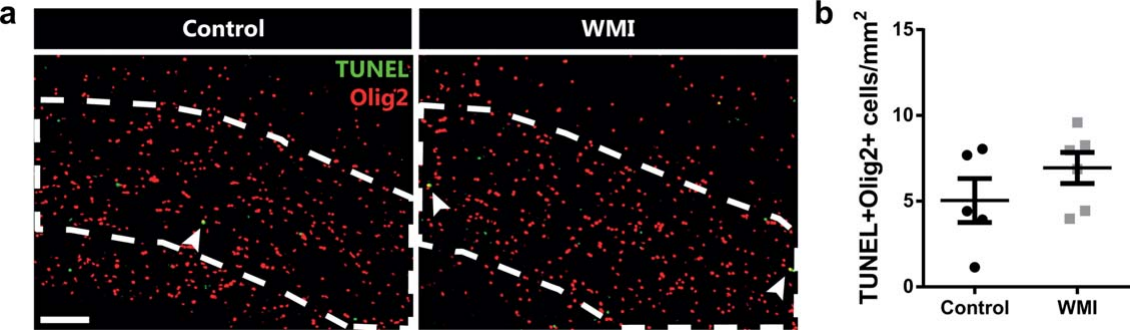
WMI is not associated with increased oligodendrocyte cell death at P5. (a) Corpus callosum (outlined) of control or WMI animals at P5 double‐stained for DNA fragmentation marker TUNEL (green) and oligodendrocyte marker Olig2 (red) (arrowheads). Scale bar: 100 μm. (b) No significant changes in the number of TUNEL^+^Olig2^+^ cells were observed in the corpus callosum of control (*n* = 5) vs. WMI animals (*n* = 6) [Color figure can be viewed at wileyonlinelibrary.com]

To elucidate whether fetal inflammation plus postnatal hypoxia induced oligodendrocyte cell death, we performed a TUNEL assay on sections of P5 rats that were stained for oligodendrocyte marker Olig2. In the corpus callosum, we sporadically observed TUNEL^+^Olig2^+^ cells in both control and WMI animals, but no differences were observed between the groups (*p* = .246) (Figure 3). Taken together, these results indicate that impaired myelination in WMI animals results from arrested oligodendrocyte maturation, rather than oligodendrocyte‐specific cell death.

### 3.3 WMI is associated with increased activation of microglia and astrocytes

**Figure 4:**
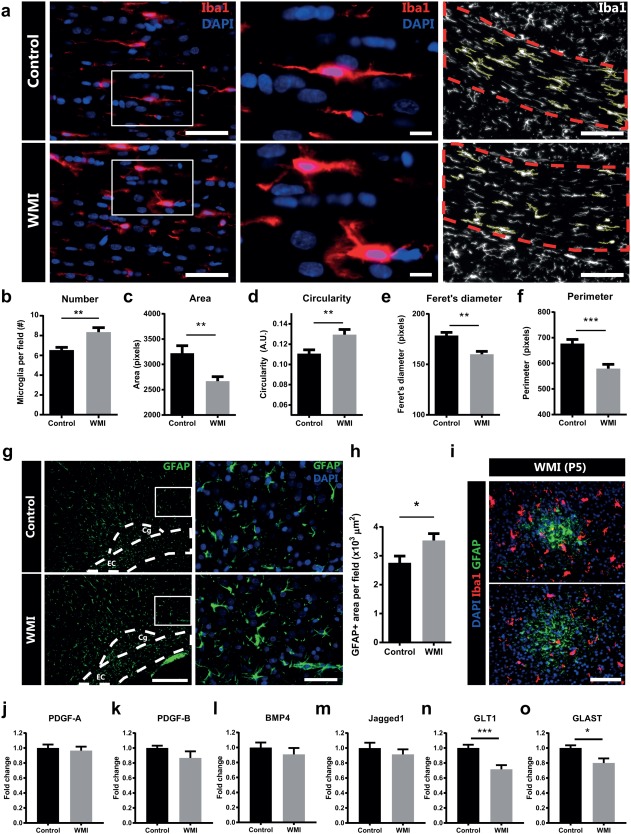
Microglia and astrocyte activity in WMI animals. (a) Left and middle: corpus callosum of P18 control vs. WMI animals stained for microglia marker Iba1 (red) and nuclear marker DAPI (blue). Middle panels show higher magnification of left panels. Right: the right panel demonstrates how the morphology of microglia (Iba1 staining of P18 animals in white) in the corpus callosum was assessed. The red line marks the corpus callosum. Yellow lines highlight included microglia. Scale bars: 50 μm (left), 10 μm (middle), 100 μm (right). (b) The number of Iba1^+^ microglia per field was increased in WMI animals compared with control animals at P18. (c‐f) For each animal, several morphological aspects of all measured microglia in the corpus callosum were averaged and recorded. Increased circularity and decreased area, Feret's diameter and perimeter indicate a more pro‐inflammatory phenotype in WMI animals at P18 (Zanier et al., 2015). (g) GFAP staining along the white matter of P18 control and WMI rats (GFAP: green; DAPI: blue; Cg: cingulum; EC: external capsule). Scale bars: 200 μm (left), 50 μm (right). (h) Quantification of GFAP^+^ area reveals increased GFAP reactivity in WMI animals compared with control animals at P18. (a)‐(h): control (*n* = 10) vs. WMI animals (*n* = 17). (i) Staining for microglia marker Iba1 (red), astrocyte marker GFAP (green) and DAPI (blue) reveals cortical GFAP^+^ patches in P5 animals. Scale bar: 100 μm. (j‐o) Compared with control rats, WMI animals do not show changes in cerebral mRNA expression of PDGF‐A, PDGF‐B, BMP4 or Jagged1 at P5. WMI animals show a decrease in cerebral mRNA expression of GLT‐1 and GLAST, indicating that reactive astrocytes may contribute to WMI pathology by attenuating their glutamate reuptake (control: *n* = 12; WMI: *n* = 13). *: *p* < .05; **: *p* < .01; ***: *p* < .001 [Color figure can be viewed at wileyonlinelibrary.com]

To investigate the activation of different glia populations, sections of P5 and P18 animals were stained for the microglia marker Iba1 and the astrocyte marker GFAP. In the corpus callosum of WMI animals, a significant increase in the number of microglia was observed (*p* = .008) (Figure 4a,b). A more detailed analysis of microglia morphology, including measures for circularity and perimeter, revealed a more proinflammatory, amoeboid morphology of these cells in WMI animals compared with controls (Figure 4c–f). Similar observations were made in the cortex of WMI animals in comparison to control rats, albeit somewhat less pronounced (Supporting Information Figure S4). In addition to microglia activity, we assessed astrocyte reactivity at P18, medial to the cingulum. We observed a significant increase in GFAP^+^ area in WMI animals compared with control pups (*p* = .041) (Figure 4g,h). Interestingly, at P5 we observed pathological GFAP^+^ gliosis patches in varying cortical areas, in 30% of the WMI animals (Figure 4i). GFAP^+^ gliosis patches were not observed in control animals at P5, nor were they observed in WMI animals at P18 or later, indicating that these GFAP^+^ patches disappear over time.

To investigate in what way reactive astrocytes may contribute to the pathology observed in WMI animals, one day after hypoxia we measured cerebral mRNA expression levels of genes that were previously associated with astrocyte‐mediated myelination impairments. Expression levels of platelet derived growth factor (PDGF)‐A, PDGF‐B, bone morphogenic protein (BMP)4 and Jagged1 (*p* = .631; *p* = .176; *p* = .413; *p* = .393, respectively) were not affected by WMI as compared with control animals (Figure 4j–m). Interestingly, WMI animals showed significantly reduced mRNA expression levels of both glutamate transporter (GLT)‐1 (*p* = .0005) and glutamate aspartate transporter (GLAST) (*p* = .012) compared with control rats (Figure 4n,o). These findings provide an interesting lead regarding the mechanisms underlying WMI resulting from fetal inflammation plus postnatal hypoxia.

### 3.4 A negative correlation between cortical FA values and MBP staining

**Figure 5:**
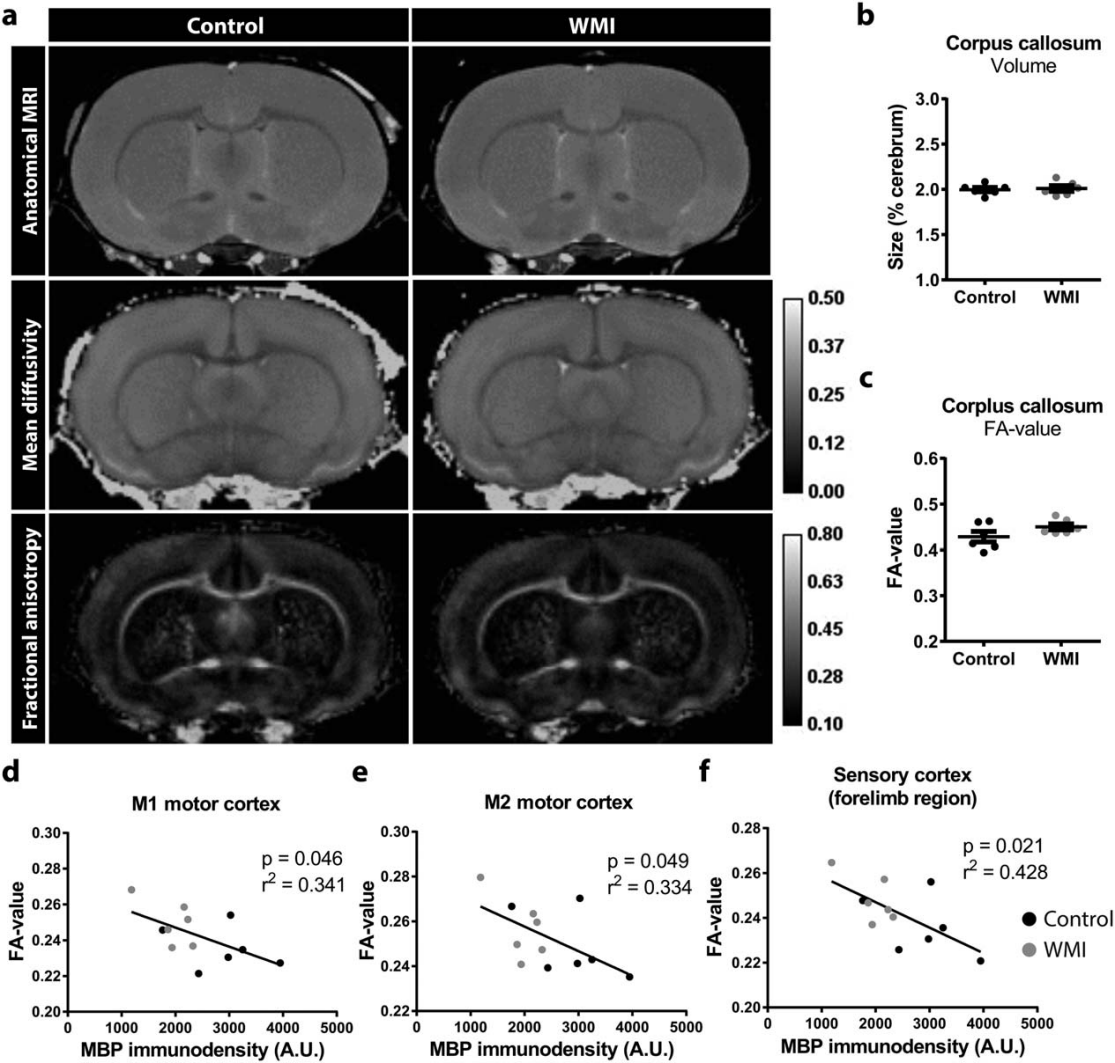
MRI scans reveal no gross neuroanatomical changes in WMI animals. (a) Representative anatomical images (top), mean diffusivity (middle) and FA (bottom) maps from control and WMI animals reveal no gross changes in neuroanatomy, nor in diffusion parameters. (b,c) WMI animals did not show a reduction in the relative size or FA values of the corpus callosum compared with controls (*p* = .771, *p* = .139, respectively) (*n* = 6 per group). (d‐f) Negative correlations were observed between cortical MBP immunostaining and FA values in the M1 cortex (*R* ^2^ = 0.341; *p* = .046), the M2 cortex (*R* ^2^ = 0.334; *p* = .049) and the forelimb region of the sensory cortex (*R* ^2^ = 0.428; *p* = .021)

Diffuse WMI in preterm infants is associated with reduced FA values, which are predictive of impaired cognitive outcome (Ment, Hirtz, & Huppi, 2009; Thompson et al., 2016; van Kooij et al., 2012). Using postmortem MRI, we assessed the size, as well as diffusivity and FA parameters of various brain regions at P30. WMI animals did not show any gross neuroanatomical changes (Figure 5a). Furthermore, no differences between WMI animals and controls were observed in the size of predefined ROIs, nor in the FA values and mean diffusivity values of these regions, including the corpus callosum (Supporting Information Table S1; Figure 5b,c). No significant changes were observed in relative white matter volumes (control: 20.4% ± 1.1%; WMI: 18.7% ± 0.9%), however we observed a negative correlation between FA values and myelination as determined by MBP immunodensity in several cortical areas (Figure 5d–f).

### 3.5 Functional consequences of WMI include impaired motor skills and autism‐like behavior

**Figure 6:**
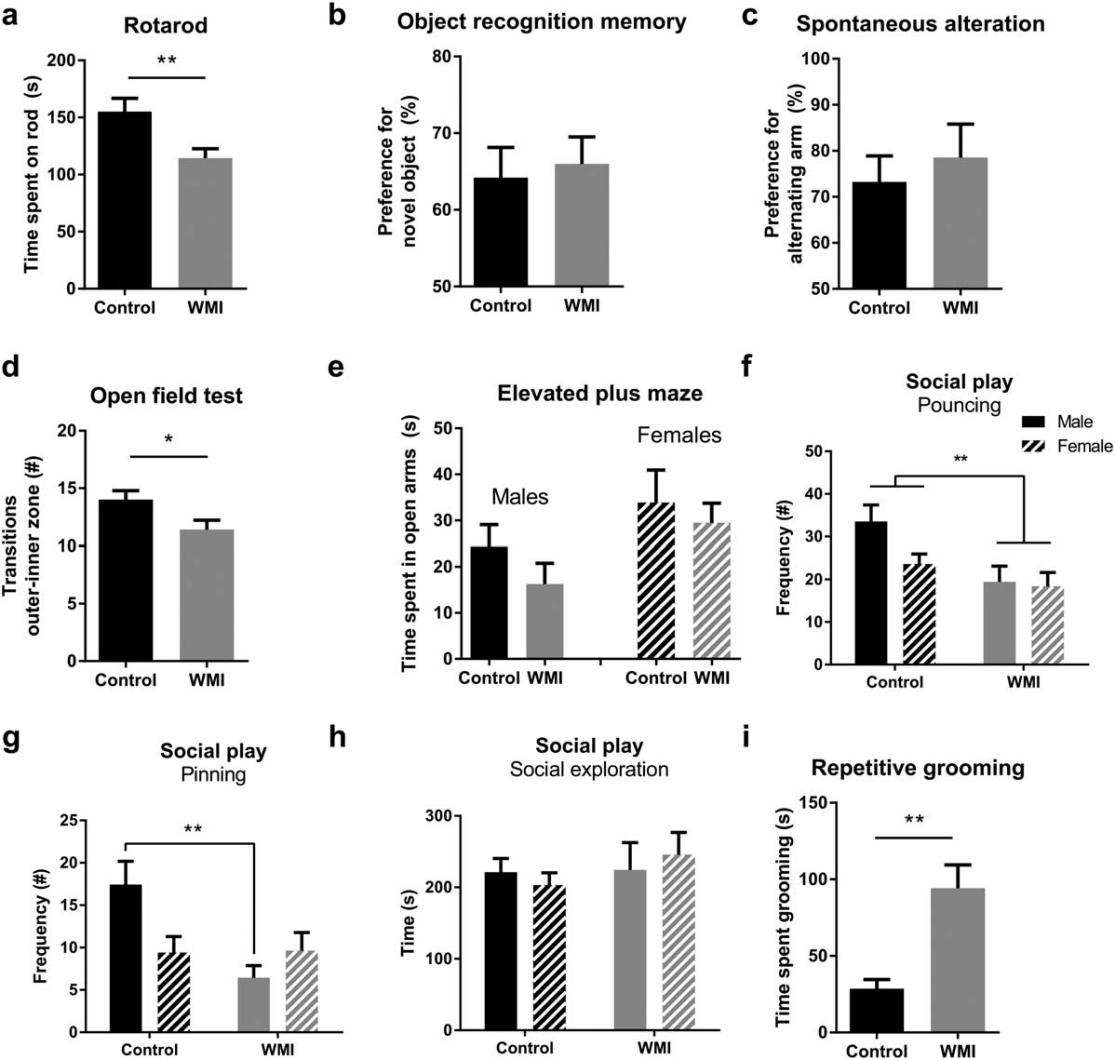
Functional consequences of combined fetal inflammation and postnatal hypoxia. (a) WMI animals (n = 12) performed worse compared with control animals (*n* = 11) on the rotarod (RTR) task, indicating impaired motor coordination. (b) No changes between control (*n* = 13) and WMI animals (*n* = 15) in object recognition memory were observed on the novel object recognition task. (c) No differences were observed between control (*n* = 9) and WMI animals (*n* = 9) on delayed spontaneous alteration in a T‐maze. (d) Compared with control animals (*n* = 12), WMI animals (*n* = 15) entered the inner zone of the open field less frequently. (e) On the elevated plus maze, we observed significant sex differences. However, no significant differences were found in male control (*n* = 14) vs WMI (*n* = 12) animals or in female control (*n* = 15) vs WMI (*n* = 7) animals in time spent in the open arms of the EPM. (f) During analysis of social play behavior, we observed a significant main effect of WMI on pouncing behavior. There was no significant effect of sex, nor was there an interaction between sex and WMI (males: *n* = 14, *n* = 11; females: *n* = 15, *n* = 8). (g) No significant differences were observed between males and females on pinning behavior. However, we observed a significant interaction between sex and WMI. Multiple comparisons revealed that WMI reduced pinning behavior in males (control *n* = 14; WMI *n* = 11), but not in females (control *n* = 15; WMI *n* = 8). (h) Social exploration was not affected by WMI in both males (*n* = 8 couples; *n* = 6 couples) and females (*n* = 8 couples; *n* = 4 couples). (i) WMI animals (*n* = 10) spent more time grooming, compared with control animals (*n* = 9). *: *p* < .05; **: *p* < .01

To assess functional consequences of WMI in rats, animals were subjected to a number of behavioral tests. First of all, WMI animals demonstrated impaired motor performance on the rotarod task (*p* = .009) (Figure 6a). Second, no changes were observed in object recognition memory (*p* = .736) or working memory (*p* = .568) (Figure 6b,c), as assessed by performance on the NORT and delayed spontaneous alteration on the T‐maze, respectively. Third, anxiety‐related behavior was assessed on the open field task and the EPM task. In the open field task, WMI animals entered the inner zone of the arena less frequently compared with control animals (*p* = .035), which is a sign of anxiety‐like behavior (Figure 6d). On the EPM, we observed a significant main effect of sex, indicating that female animals spent more time in the open arms compared with males (*p* = .031). However, there was no significant main effect of WMI, nor was there a significant interaction between sex and WMI (Figure 6e). On both the open field task and the EPM, no differences in locomotor activity were observed between control and WMI animals (data not shown).

Preterm infants are at risk to develop ASD (Dudova et al., 2014; Johnson et al., 2010; Joseph et al., 2017; Kuzniewicz et al., 2014; Limperopoulos et al., 2008; Pritchard et al., 2016; Treyvaud et al., 2013). To assess autism‐like behavior, we studied social play behavior and grooming behavior of the animals. Social functioning was assessed at the age of 4 weeks by analyzing social play behavior after 2.5 hr of social isolation. Several differences were observed in play behavior. First, WMI animals demonstrated reduced pouncing behavior (main effect WMI: *p* = .009), regardless of sex (main effect sex: *p* = .124; interaction: 0.214) (Figure 6f). For pinning behavior, a statistically significant interaction was observed between sex and WMI (*p* = .019): compared with control animals, males with WMI showed reduced pinning behavior (*p* = .002), whereas this effect was not observed in females (*p* > .999) (Figure 6g). General social exploration (e.g., sniffing) was not affected by WMI in both sexes (*p* = .399) (Figure 6h), indicating a specific effect on play behavior. Additionally, WMI animals showed a threefold increase in repetitive grooming behavior compared with control animals (*p* = .002) (Figure 6i), which is associated with autism‐like behavior (Kalueff et al., 2016).

## 4 DISCUSSION

In the present study, we assessed the functional and neuroanatomical consequences of a multiple hit model of diffuse WMI in rats, by behavioral paradigms, MRI and immunostainings. Whereas exposure to a single hit did not induce myelination deficits, rats exposed to both fetal inflammation and postnatal hypoxia displayed a pathology closely resembling that of preterm infants with diffuse WMI, including delayed myelination and impaired oligodendrocyte differentiation. At P5, 30% of the WMI animals showed cortical gliosis patches that disappear over time and might originate from microscopic ischemic or hemorrhagic events, or from blood‐brain barrier leakage. Furthermore, a more amoeboid morphology of microglia was observed in both the white and gray matter, indicating a primed or pro‐inflammatory phenotype of these cells, which may contribute to the hampered maturation of oligodendrocytes (Pang, Zheng, Fan, Rhodes, & Cai, 2007). Additionally, we observed a widespread increase in microglia numbers. Considering microglia are highly proliferative cells that undergo clonal expansion after injury (Askew et al., 2017; Tay et al., 2017), it is likely that increased numbers of Iba1+ cells are a consequence of increased proliferation (Umekawa, Oman, Han, Ikeda, & Blomgren, 2015), although the possibilities of microglia migrating from distant brain regions or infiltrating macrophages cannot be excluded. At P18 WMI animals showed increased immunoreactivity of GFAP in comparison to control brains, indicating the presence of reactive astrocytes. To investigate how astrocyte reactivity may contribute to WMI pathology, we analyzed expression levels of several genes that previously have been associated with astrocyte‐mediated myelination impairments. Astrocytes are an important source of PDGF, which inhibits OPC differentiation (Gard, Burrell, Pfeiffer, Rudge, & Williams, 1995; Silberstein, De Simone, Levi, & Aloisi, 1996). Furthermore, reactive astrocytes have been associated with increased expression of BMP4 and Jagged1, two factors that inhibit OPC differentiation (Hammond et al., 2014; Reid et al., 2012; Wang et al., 2011). No changes were observed in the expression of PDGF, BMP4 or Jagged1 in brains of WMI animals versus controls. Another important function of astrocytes is glutamate reuptake through glutamate transporters GLT‐1 and GLAST, which we showed to be downregulated in WMI animals. These results are in line with earlier findings of transiently reduced expression of GLT‐1 and GLAST after chronic hypoxia (Raymond, Li, Mangin, Huntsman, & Gallo, 2011). Deficiency in glutamate uptake causes increased availability of glutamate, and hampers proper development of OPCs through AMPA/NMDA receptor‐mediated excitotoxicity (Volpe, Kinney, Jensen, & Rosenberg, 2011). These findings provide an interesting lead regarding the mechanisms through which reactive astrocyte may contribute to WMI, but more detailed analyses are needed to directly relate these findings to astrogliosis and to definitively assess the contribution of excitotoxicity to WMI pathology.

In WMI rats, cortical myelination was reduced up to P30. Impeded maturation of oligodendrocytes likely underlies the impaired myelination in WMI animals, as indicated by increased numbers of proliferating OPCs and reduced proportion of more mature CNPase‐expressing oligodendrocytes at P18. Also, we did not find evidence of increased oligodendrocyte cell death at P5. In addition, our data indicate that at least to the extent of the sensory cortex, white matter development is delayed rather than permanently damaged since myelination deficits seem largely restored by the age of P69. Interestingly, long‐term changes in oligodendrocyte maturation remained present at P69, as indicated by the lower number of proliferating OPCs in the corpus callosum and the percentage of CNPase^+^Olig2^+^ cells in the cortex. An explanation for the reduced number of proliferating OPCs at P69 might be that due to increased proliferation during development (P18) the OPC pool is expanded, resulting in a downregulation of proliferation at later ages (P69). This would be in line with findings of increased numbers of Olig2^+^ cells in postmortem WMI brain tissue (Buser et al., 2012). Despite partially restored MBP levels at P69, the possibilities cannot be excluded that myelination in brain regions other than the sensory cortex do not fully restore to control levels, or that physiological properties of cortical myelin (e.g., myelin sheath thickness) remain affected at later ages. Alterations in these physiological properties may account for the impaired functional outcome that remains present in older rats, despite restored MBP levels. Myelin‐independent mechanisms may also contribute to impaired functional outcome at later ages. For example, reductions in gamma‐aminobutyric acid (GABA)‐expressing neurons were observed in postmortem tissue of preterm infants with perinatal brain injury (Robinson et al., 2006) and damage to GABAergic interneurons has been implicated in the pathophysiology of hypoxia‐induced diffuse WMI in mice (Komitova et al., 2013; Zonouzi et al., 2015).

On a behavioral level, WMI animals displayed impaired motor performance, anxiety‐like behavior and signs of autism‐like behavior, i.e. repetitive self‐grooming and a reduction in social play behavior. In line with these findings, recent clinical studies revealed an association between diffuse WMI and motor abnormalities in preterm infants (Guo et al., 2017; Peyton et al., 2016, 2017). Also, multiple studies have demonstrated that preterm infants are at risk to develop ASD later in life (Dudova et al., 2014; Johnson et al., 2010; Joseph et al., 2017; Kuzniewicz et al., 2014; Limperopoulos et al., 2008; Pritchard et al., 2016; Treyvaud et al., 2013). Although a clear association exists between preterm birth and ASD, and between white matter integrity and ASD (Ameis & Catani, 2015; Ameis et al., 2016; Catani et al., 2016; Fitzgerald, Gallagher, & McGrath, 2016; Vogan et al., 2016), the relationship between preterm birth‐related WMI and ASD remains poorly investigated. One study showed an association between *cystic* white matter lesions and ASD in preterm infants (Ure et al., 2016). However, the link between ASD and noncystic, diffuse white matter abnormalities in preterm infants has, to our knowledge, not been studied. Furthermore, both clinical and experimental evidence indicate that inflammation *in utero* is associated with ASD (reviewed in (Estes & McAllister, 2016)). Considering our current data showing signs of autism‐like behavior in rats with diffuse WMI, it would be interesting to further investigate whether ASD in preterm infants is directly related to diffuse changes in white matter microstructure. We observed that social play behavior was affected in a more pronounced manner in male WMI rats, compared with females (i.e. males showed attenuated pinning and pouncing, females showed reduced pouncing). This observation seems to reflect the clinical situation, where male gender is an important predictor for ASD in the preterm population (Johnson et al., 2010; Limperopoulos et al., 2008; Stephens et al., 2012). No signs of severe impairments in object recognition memory and working memory were detected. This is in line with the clinical situation, in which preterm birth is generally associated with subtle cognitive impairments, rather than severe mental retardation (Burnett, Scratch, & Anderson, 2013; Nosarti & Froudist‐Walsh, 2016). To study whether rats exposed to fetal inflammation and postnatal hypoxia have impaired cognitive abilities, more complex behavioral tasks should be performed in the future.

Postmortem MRI revealed no significant differences in MD or FA values in WMI animals compared with control animals at P30. These data are similar to observations made in a rat model of fetal growth restriction, where reductions in FA values were observed at P10, but which returned to normal, or even regionally increased FA values at P21 (Rideau Batista Novais et al., 2016). When interpreting data from these rodent models of neonatal brain injury, it should be considered that such findings are in contrast with the general view that WMI in preterm infants is associated with reduced FA values in white matter tracts, which predict adverse outcome (Allin et al., 2011; van Kooij et al., 2012; Ment et al., 2009). However, various imaging studies demonstrated that preterm birth can also cause regionally increased FA values (Brossard‐Racine et al., 2017; Padilla et al., 2014; Travis, Adams, Ben‐Shachar, & Feldman, 2015). Together, these data indicate that FA findings in developing mammals are highly time‐ and region‐dependent. This should be taken into account when interpreting MRI data, especially in rodent models considering the high restorative capacity of white matter microstructure that has been observed in various models of neonatal brain injury (Rideau Batista Novais et al., 2016). Additionally, we observed a negative correlation between FA values and myelination as indicated by MBP immunostaining in several cortical areas. Although a negative correlation seems in contrast with expected lower FA values in WMI, increased FA values in the cortex might be explained by reduced arborization of myelinated axons, i.e. less complex myelination of axons in the cortex.

We demonstrated that combined fetal inflammation and postnatal hypoxia induces a relevant pattern of diffuse WMI. As in the clinical situation, the resulting pattern of injury is relatively subtle. A possible limitation of the model is that in contrast to the clinical situation, we did not observe gross neuroanatomical changes such as increased ventricle size and thinning of the corpus callosum. Despite this limitation, we propose that the combination of fetal inflammation and postnatal hypoxia can be used as a novel translational model to investigate underlying mechanisms, as well as effects of novel treatment options on oligodendrocyte maturation, myelination, glial cell activation and related behavioral abnormalities. Previously, various rodent models have been proposed to study perinatal diffuse WMI (Back, & Rosenberg, 2014; Silbereis, Huang, Back, & Rowitch, 2010; van Tilborg et al., 2016). Hypoxic rearing (Ganat, Soni, Chacon, Schwartz, & Vaccarino, 2002; Ment, Schwartz, Makuch, & Stewart, 1998; Scafidi et al., 2014), transient hyperoxia (Gerstner et al., 2008), maternal inflammation (Rousset et al., 2006), and postnatal systemic inflammation (Favrais et al., 2011) have been demonstrated to affect myelination without causing cystic lesions. These models mimic certain aspects of the distress encountered by preterm infants and can be used as a model for specific populations of preterm infants that are exposed to e.g., respiratory deficits, ventilation therapy or maternal/postnatal infections. Our multiple‐hit model distinguishes itself from the previously mentioned models, as it takes in to account the multifactorial etiology of diffuse WMI in preterm infants (Korzeniewski et al., 2014; Leviton et al., 2013), by applying two subtle insults during a peak of immature oligodendrocytes residing in the developing brain (Salmaso, Jablonska, Scafidi, Vaccarino, & Gallo, 2014; Semple, Blomgren, Gimlin, Ferriero, & Noble‐Haeusslein, 2013). Similarly, it was shown that the combination of LPS exposure at P3 plus hyperoxia for 24 hr at P6 negatively affects white matter development in mice (Brehmer et al., 2012). Our data show that the combination of both the inflammatory and the hypoxic insult is required to induce white matter deficits. It has been suggested previously that early inflammatory insults sensitize the brain to subsequent injurious events (Kaindl et al., 2009); Van Steenwinckel et al., 2014). Interestingly, the intrauterine environment is relatively hypoxic compared with extra‐uterine conditions. Yet, due to immature and often diseased respiratory, vascular and immune systems, preterm infants are often exposed to unstable oxygen saturation levels (Van Tilborg et al., 2016). Although optimal oxygen saturation levels in preterm infants are still an important topic of debate (Castillo et al., 2008; Lakshminrusimha, Manja, Mathew, & Suresh, 2015), in case of hypoxia infants are often treated with ventilation therapy, receiving supplemental oxygen which further contributes to large fluctuations in oxygen levels and which has been associated with increased levels of harmful oxidative stress (Perrone, Bracciali, Di Virgilio, & Buonocore, 2017). Oxygen instability has been used in a variety of neonatal rodent models to mimic preterm birth‐related brain injury. For example, hyperoxic and intermittent hypoxic periods in neonatal rodents have been shown to cause brain damage (Darnall et al., 2017; Gerstner et al., 2008). Furthermore, hypoxic rearing by placing complete litters with nursing mothers at 9–11% O_2_ for >7 days starting at P3 causes myelination deficits (Ment et al., 1998; Scafidi et al., 2014). In comparison, the hypoxic insult (140 min at 8% O_2_) used in the present study is relatively subtle and short‐lived but due to an earlier sensitizing inflammatory insult, the detrimental effects may be aggravated. Considering the various types of inflammatory insults and oxygen fluctuations that preterm infants often encounter, the combination of two (relatively mild) hits is highly relevant for clinical translation.

Taken together, combined fetal inflammation and postnatal hypoxia induces clinically relevant diffuse white matter pathology in neonatal rats in terms of neuroanatomy and functional outcome. This model can contribute to the investigation of novel treatment strategies aimed at attenuating neuroinflammation and promoting oligodendrocyte differentiation and myelination, which can lead to development of new, desperately needed therapies to combat diffuse WMI in preterm infants.

## CONFLICTS OF INTEREST

The authors declare no conflict of interest.

## Supporting information

Supporting Information Figure 1.

Supporting Information Figure 2.

Supporting Information Figure 3.

Supporting Information Figure 4.

Supporting Information Table 1.

## References

[glia23216-bib-0001] Achterberg, E. J. , van Kerkhof, L. W. , Damsteegt, R. , Trezza, V. , & Vanderschuren, L. J. (2015). Methylphenidate and atomoxetine inhibit social play behavior through prefrontal and subcortical limbic mechanisms in rats. Journal of Neuroscience, 35, 161–169. 25568111 10.1523/JNEUROSCI.2945-14.2015 PMC4287139

[glia23216-bib-0002] Allin, M. P. , Kontis, D. , Walshe, M. , Wyatt, J. , Barker, G. J. , Kanaan, R. A. , … Nosarti, C. (2011). White matter and cognition in adults who were born preterm. PLoS One, 6, e24525. 22022357 10.1371/journal.pone.0024525 PMC3192037

[glia23216-bib-0003] Ameis, S. H. , & Catani, M. (2015). Altered white matter connectivity as a neural substrate for social impairment in Autism Spectrum Disorder. Cortex, 62, 158–181. 25433958 10.1016/j.cortex.2014.10.014

[glia23216-bib-0004] Ameis, S. H. , Lerch, J. P. , Taylor, M. J. , Lee, W. , Viviano, J. D. , Pipitone, J. , … Anagnostou, E. (2016). A diffusion tensor imaging study in children with ADHD, autism spectrum disorder, OCD, and matched controls: Distinct and non‐distinct white matter disruption and dimensional brain‐behavior relationships. The American Journal of Psychiatry, 173, 1213–1222. 27363509 10.1176/appi.ajp.2016.15111435

[glia23216-bib-0005] Askew, K. , Li, K. , Olmos‐Alonso, A. , Garcia‐Moreno, F. , Liang, Y. , Richardson, P. , … Gomez‐Nicola, D. (2017). Coupled proliferation and apoptosis maintain the rapid turnover of microglia in the adult brain. Cell Reports, 18, 391–405. 28076784 10.1016/j.celrep.2016.12.041 PMC5263237

[glia23216-bib-0006] Back, S. A. , & Miller, S. P. (2014). Brain injury in premature neonates: A primary cerebral dysmaturation disorder? Annals of Neurology, 75, 469–486. 24615937 10.1002/ana.24132 PMC5989572

[glia23216-bib-0007] Back, S. A. , & Rosenberg, P. A. (2014). Pathophysiology of glia in perinatal white matter injury. Glia, 62, 1790–1815. 24687630 10.1002/glia.22658 PMC4163108

[glia23216-bib-0008] Billiards, S. S. , Haynes, R. L. , Folkerth, R. D. , Borenstein, N. S. , Trachtenberg, F. L. , Rowitch, D. H. , … Kinney, H. C. (2008). Myelin abnormalities without oligodendrocyte loss in periventricular leukomalacia. Brain Pathology, 18, 153–163. 18177464 10.1111/j.1750-3639.2007.00107.x PMC2770329

[glia23216-bib-0009] Brehmer, F. , Bendix, I. , Prager, S. , van de Looij, Y. , Reinboth, B. S. , Zimmermanns, J. , … Gerstner, B. (2012). Interaction of inflammation and hyperoxia in a rat model of neonatal white matter damage. PLoS One, 7, e49023. 23155446 10.1371/journal.pone.0049023 PMC3498343

[glia23216-bib-0010] Brossard‐Racine, M. , Poretti, A. , Murnick, J. , Bouyssi‐Kobar, M. , McCarter, R. , du Plessis, A. J. , & Limperopoulos, C. (2017). Cerebellar Microstructural Organization is Altered by Complications of Premature Birth: A Case‐Control Study. Journal of Pediatrics, 182, 28–33.e1. doi:10.1016/j.jpeds.2016.10.034 27843009 10.1016/j.jpeds.2016.10.034 PMC13159174

[glia23216-bib-0011] Burnett, A. C. , Scratch, S. E. , & Anderson, P. J. (2013). Executive function outcome in preterm adolescents. Early Human Development, 89, 215–220. 23455604 10.1016/j.earlhumdev.2013.01.013

[glia23216-bib-0012] Buser, J. R. , Maire, J. , Riddle, A. , Gong, X. , Nguyen, T. , Nelson, K. , … Back, S. A. (2012). Arrested preoligodendrocyte maturation contributes to myelination failure in premature infants. Annals of Neurology, 71, 93–109. 22275256 10.1002/ana.22627 PMC3270934

[glia23216-bib-0013] Cai, Z. , Pan, Z. L. , Pang, Y. , Evans, O. B. , & Rhodes, P. G. (2000). Cytokine induction in fetal rat brains and brain injury in neonatal rats after maternal lipopolysaccharide administration. Pediatrric Research, 47, 64–72. 10.1203/00006450-200001000-00013 10625084

[glia23216-bib-0014] Castillo, A. , Sola, A. , Baguero, H. , Neira, F. , Alvis, R. , Deulofeut, R. , & Critz, A. (2008). Pulse oxygen saturation levels and arterial oxygen tension values in newborns receiving oxygen therapy in the neonatal intensive care unit: is 85% to 93% an acceptable range? Pediatrics, 121, 882–889. 18450890 10.1542/peds.2007-0117

[glia23216-bib-0015] Catani, M. , Dell'Acqua, F. , Budisavljevic, S. , Howells, H. , Thiebaut de Schotten, M. , Froudist‐Walsh, S. , … Murphy, D. G. (2016). Frontal networks in adults with autism spectrum disorder. Brain, 139, 616–630. 26912520 10.1093/brain/awv351 PMC4805089

[glia23216-bib-0016] Chau, V. , Brant, R. , Poskitt, K. J. , Tam, E. W. , Synnes, A. , & Miller, S. P. (2012). Postnatal infection is associated with widespread abnormalities of brain development in premature newborns. Pediatric Research, 71, 274–279. 22278180 10.1038/pr.2011.40 PMC3940469

[glia23216-bib-0017] Crowther, C. A. , Hiller, J. E. , Doyle, L. W. , Haslam, R. R. , & Australasian Collaborative Trial of Magnesium Sulfate (ACTOMg SO4) Collaborative Group (2003). Effect of magnesium sulfate given for neuroprotection before preterm birth: a randomized controlled trial. JAMA, 290, 2669–2676. 14645308 10.1001/jama.290.20.2669

[glia23216-bib-0018] Darnall, R. A. , Chen, X. , Nemani, K. V. , Sirieix, C. M. , Gimi, B. , Knoblach, S. , … Hunt, C. E. (2017). Early postnatal exposure to intermittent hypoxia in rodents is proinflammatory, impairs white matter integrity, and alters brain metabolism. Pediatric Research, 82, 164–172. 28388601 10.1038/pr.2017.102 PMC5509485

[glia23216-bib-0019] Doyle, L. W. , Crowther, C. A. , Middleton, P. , Marret, S. , & Rouse, D. (2009). Magnesium sulphate for women at risk of preterm birth for neuroprotection of the fetus. Cochrane Database of Systematic Reviews, 1. Art. No.: CD004661. doi:10.1002/14651858.CD004661.pub3. 10.1002/14651858.CD004661.pub3 19160238

[glia23216-bib-0020] Dudova, I. , Kasparova, M. , Markova, D. , Zemankova, J. , Beranova, S. , Urbanek, T. , & Hrdlicka, M. (2014). Screening for autism in preterm children with extremely low and very low birth weight. Neuropsychiatric Disease and Treatment, 10, 277–282. 24627633 10.2147/NDT.S57057 PMC3931701

[glia23216-bib-0021] Estes, M. L. , & McAllister, A. K. (2016). Maternal immune activation: Implications for neuropsychiatric disorders. Science, 353, 772–777. 27540164 10.1126/science.aag3194 PMC5650490

[glia23216-bib-0022] Favrais, G. , van de Looij, Y. , Fleiss, B. , Ramanantsoa, N. , Bonnin, P. , Stoltenburg‐Didinger, G. , … Gressens, P. (2011). Systemic inflammation disrupts the developmental program of white matter. Annals of Neurology, 70, 550–565. 21796662 10.1002/ana.22489

[glia23216-bib-0023] Fitzgerald, J. , Gallagher, L. , & McGrath, J. (2016). Widespread Disrupted White Matter Microstructure in Autism Spectrum Disorders. Journal of Autism and Developmental Disorders. doi:10.1007/s10803-016-2803-8. 10.1007/s10803-016-2803-8 27207090

[glia23216-bib-0024] Fonck, E. , Feigl, G. G. , Fasel, J. , Sage, D. , Unser, M. , Rufenacht, D. A. , & Stergiopulos, N. (2009). Effect of aging on elastin functionality in human cerebral arteries. Stroke, 40, 2552–2556. 19478233 10.1161/STROKEAHA.108.528091

[glia23216-bib-0025] Ganat, Y. , Soni, S. , Chacon, M. , Schwartz, M. L. , & Vaccarino, F. M. (2002). Chronic hypoxia up‐regulates fibroblast growth factor ligands in the perinatal brain and induces fibroblast growth factor‐responsive radial glial cells in the sub‐ependymal zone. Neuroscience, 112, 977–991. 12088755 10.1016/s0306-4522(02)00060-x

[glia23216-bib-0026] Gano, D. , Ho, M. L. , Partridge, J. C. , Glass, H. C. , Xu, D. , Barkovich, A. J. , & Ferriero, D. M. (2016). Antenatal exposure to magnesium sulfate is associated with reduced cerebellar hemorrhage in preterm newborns. Journal of Pediatrics, 178, 68–74. 27453378 10.1016/j.jpeds.2016.06.053 PMC5085851

[glia23216-bib-0027] Gard, A. L. , Burrell, M. R. , Pfeiffer, S. E. , Rudge, J. S. , & Williams, W. C. , 2nd. (1995). Astroglial control of oligodendrocyte survival mediated by PDGF and leukemia inhibitory factor‐like protein. Development, 121, 2187–2197. 7635062 10.1242/dev.121.7.2187

[glia23216-bib-0028] Gerstner, B. , DeSilva, T. M. , Genz, K. , Armstrong, A. , Brehmer, F. , Neve, R. L. , … Rosenberg, P. A. (2008). Hyperoxia causes maturation‐dependent cell death in the developing white matter. Journal of Neuroscience, 28, 1236–1245. 18234901 10.1523/JNEUROSCI.3213-07.2008 PMC4305399

[glia23216-bib-0029] Guo, T. , Duerden, E. G. , Adams, E. , Chau, V. , Branson, H. M. , Chakravarty, M. M. , … Miller, S. P. (2017). Quantitative assessment of white matter injury in preterm neonates: Association with outcomes. Neurology, 88, 614–622. 28100727 10.1212/WNL.0000000000003606 PMC5317385

[glia23216-bib-0030] Hammad, A. M. , Althobaiti, Y. S. , Das, S. C. , & Sari, Y. (2017). Effects of repeated cocaine exposure and withdrawal on voluntary ethanol drinking, and the expression of glial glutamate transporters in mesocorticolimbic system of P rats. Molecular and Cellular Neuroscience, 82, 58–65. 28442364 10.1016/j.mcn.2017.04.008 PMC5533096

[glia23216-bib-0031] Hammond, T. R. , Gadea, A. , Dupree, J. , Kerninon, C. , Nait‐Oumesmar, B. , Aguirre, A. , & Gallo, V. (2014). Astrocyte‐derived endothelin‐1 inhibits remyelination through notch activation. Neuron, 81, 588–602. 24507193 10.1016/j.neuron.2013.11.015 PMC3935216

[glia23216-bib-0032] Haynes, R. L. , Folkerth, R. D. , Keefe, R. J. , Sung, I. , Swzeda, L. I. , Rosenberg, P. A. , … Kinney, H. C. (2003). Nitrosative and oxidative injury to premyelinating oligodendrocytes in periventricular leukomalacia. Journal of Neuropathology and Experimental Neurology, 62, 441–450. 12769184 10.1093/jnen/62.5.441

[glia23216-bib-0033] Hotaling, N. A. , Bharti, K. , Kriel, H. , & Simon, C. G., Jr. (2015). DiameterJ: A validated open source nanofiber diameter measurement tool. Biomaterials, 61, 327–338. 26043061 10.1016/j.biomaterials.2015.05.015 PMC4492344

[glia23216-bib-0034] Jenkinson, M. , Beckmann, C. F. , Behrens, T. E. , Woolrich, M. W. , & Smith, S. M. (2012). Fsl. Neuroimage, 62, 782–790. 21979382 10.1016/j.neuroimage.2011.09.015

[glia23216-bib-0035] Johnson, S. , Hollis, C. , Kochhar, P. , Hennessy, E. , Wolke, D. , & Marlow, N. (2010). Autism spectrum disorders in extremely preterm children. Journal of Pediatrics, 156, 525–531. e522. 20056232 10.1016/j.jpeds.2009.10.041

[glia23216-bib-0036] Jordan, R. (2003). Social play and autistic spectrum disorders: A perspective on theory, implications and educational approaches. Autism, 7, 347–360. 14678675 10.1177/1362361303007004002

[glia23216-bib-0037] Joseph, R. M. , O'Shea, T. M. , Allred, E. N. , Heeren, T. , Hirtz, D. , Paneth, N. , … Kuban, K. C. (2017). Prevalence and associated features of autism spectrum disorder in extremely low gestational age newborns at age 10 years. Autism Research, 10, 224–232. 27220677 10.1002/aur.1644 PMC5123971

[glia23216-bib-0038] Kaindl, A. M. , Favrais, G. , & Gressens, P. (2009). Molecular mechanisms involved in injury to the preterm brain. Journal of Child Neurology, 24, 1112–1118. https://doi.org/10.1177/0883073809337920 19605776 10.1177/0883073809337920 PMC3743549

[glia23216-bib-0039] Kalueff, A. V. , Stewart, A. M. , Song, C. , Berridge, K. C. , Graybiel, A. M. , & Fentress, J. C. (2016). Neurobiology of rodent self‐grooming and its value for translational neuroscience. Nature Reviews Neuroscience, 17, 45–59. 26675822 10.1038/nrn.2015.8 PMC4840777

[glia23216-bib-0040] Komitova, M. , Xenos, D. , Salmaso, N. , Tran, K. M. , Brand, T. , Schwartz, M. L. , … Vaccarino, F. M. (2013). Hypoxia‐induced developmental delays of inhibitory interneurons are reversed by environmental enrichment in the postnatal mouse forebrain. Journal of Neuroscience, 33, 13375–13387. 23946395 10.1523/JNEUROSCI.5286-12.2013 PMC3742925

[glia23216-bib-0041] van Kooij, B. J. , de Vries, L. S. , Ball, G. , van Haastert, I. C. , Benders, M. J. , Groenendaal, F. , & Counsell, S. J. (2012). Neonatal tract‐based spatial statistics findings and outcome in preterm infants. American Journal of Neuroradiology, 33, 188–194. 21998101 10.3174/ajnr.A2723 PMC7966173

[glia23216-bib-0042] Korzeniewski, S. J. , Romero, R. , Cortez, J. , Pappas, A. , Schwartz, A. G. , Kim, C. J. , … Hassan, S. S. (2014). A “multi‐hit” model of neonatal white matter injury: cumulative contributions of chronic placental inflammation, acute fetal inflammation and postnatal inflammatory events. Journal of Perinatal Medicine, 42, 731–743. 25205706 10.1515/jpm-2014-0250 PMC5987202

[glia23216-bib-0043] Kuzniewicz, M. W. , Wi, S. , Qian, Y. , Walsh, E. M. , Armstrong, M. A. , & Croen, L. A. (2014). Prevalence and neonatal factors associated with autism spectrum disorders in preterm infants. Journal of Pediatrics, 164, 20–25. 24161222 10.1016/j.jpeds.2013.09.021

[glia23216-bib-0044] Lakshminrusimha, S. , Manja, V. , Mathew, B. , & Suresh, G. K. (2015). Oxygen targeting in preterm infants: a physiological interpretation. Journal of Perinatology, 35, 8–15. 25357098 10.1038/jp.2014.199 PMC4281291

[glia23216-bib-0045] Leviton, A. , Fichorova, R. N. , O'Shea, T. M. , Kuban, K. , Paneth, N. , Dammann, O. , & Allred, E. N. ELGAN Study Investigators (2013). Two‐hit model of brain damage in the very preterm newborn: small for gestational age and postnatal systemic inflammation. Pediatric Research, 73, 362–370. 23364171 10.1038/pr.2012.188 PMC3642985

[glia23216-bib-0046] Limperopoulos, C. , Bassan, H. , Sullivan, N. R. , Soul, J. S. , Robertson, R. L., Jr. , Moore, M. , … du Plessis, A. J. (2008). Positive screening for autism in ex‐preterm infants: Prevalence and risk factors. Pediatrics, 121, 758–765. 18381541 10.1542/peds.2007-2158 PMC2703587

[glia23216-bib-0047] Ment, L. R. , Hirtz, D. , & Huppi, P. S. (2009). Imaging biomarkers of outcome in the developing preterm brain. The Lancet Neurology, 8, 1042–1055. https://doi.org/10.1016/S1474-4422(09)70257-1. 19800293 10.1016/S1474-4422(09)70257-1

[glia23216-bib-0048] Ment, L. R. , Schwartz, M. , Makuch, R. W. , & Stewart, W. B. (1998). Association of chronic sublethal hypoxia with ventriculomegaly in the developing rat brain. Brain Research. Developmental Brain Research, 111, 197–203. 9838111 10.1016/s0165-3806(98)00139-4

[glia23216-bib-0049] Nosarti, C. , & Froudist‐Walsh, S. (2016). Alterations in development of hippocampal and cortical memory mechanisms following very preterm birth. Developmental Medicine & Child Neurology, 58(Suppl 4), 35–45. 27027606 10.1111/dmcn.13042 PMC4819886

[glia23216-bib-0050] Padilla, N. , Junque, C. , Figueras, F. , Sanz‐Cortes, M. , Bargallo, N. , Arranz, A. , … Gratacos, E. (2014). Differential vulnerability of gray matter and white matter to intrauterine growth restriction in preterm infants at 12 months corrected age. Brain Research, 1545, 1–11. 24361462 10.1016/j.brainres.2013.12.007

[glia23216-bib-0051] Pang, Y. , Zheng, B. , Fan, L. W. , Rhodes, P. G. , & Cai, Z. (2007). IGF‐1 protects oligodendrocyte progenitors against TNFalpha‐induced damage by activation of PI3K/Akt and interruption of the mitochondrial apoptotic pathway. Glia, 55, 1099–1107. 17577243 10.1002/glia.20530

[glia23216-bib-0052] Perrone, S. , Bracciali, C. , Di Virgilio, N. , & Buonocore, G. (2017). Oxygen use in neonatal care: A two‐edged sword. Frontiers in Pediatrics, 4, 143 https://doi.org/10.3389/fped.2016.00143. 28119904 10.3389/fped.2016.00143 PMC5220090

[glia23216-bib-0053] Peyton, C. , Yang, E. , Kocherginsky, M. , Adde, L. , Fjortoft, T. , Stoen, R. , … Msall, M. E. (2016). Relationship between white matter pathology and performance on the General Movement Assessment and the test of infant motor performance in very preterm infants. Early Human Development, 95, 23–27. 26925933 10.1016/j.earlhumdev.2016.01.017

[glia23216-bib-0054] Peyton, C. , Yang, E. , Msall, M. E. , Adde, L. , Stoen, R. , Fjortoft, T. , … Drobyshevsky, A. (2017). White matter injury and general movements in high‐risk preterm infants. American Journal of Neuroradiology, 38, 162–169. 27789448 10.3174/ajnr.A4955 PMC7963672

[glia23216-bib-0055] Pritchard, M. A. , de Dassel, T. , Beller, E. , Bogossian, F. , Johnston, L. , Paynter, J. , … Scott, J. (2016). Autism in toddlers born very preterm. Pediatrics, 137, e20151949. 26798043 10.1542/peds.2015-1949

[glia23216-bib-0056] Procianoy, R. S. , & Silveira, R. C. (2012). Association between high cytokine levels with white matter injury in preterm infants with sepsis. Pediatric Critical Care Medicine, 13, 183–187. 21666535 10.1097/PCC.0b013e3182231074

[glia23216-bib-0057] Raymond, M. , Li, P. , Mangin, J. M. , Huntsman, M. , & Gallo, V. (2011). Chronic perinatal hypoxia reduces glutamate‐aspartate transporter function in astrocytes through the Janus kinase/signal transducer and activator of transcription pathway. Journal of Neuroscience, 31, 17864–17871. 22159101 10.1523/JNEUROSCI.3179-11.2011 PMC3278804

[glia23216-bib-0058] Reid, M. V. , Murray, K. A. , Marsh, E. D. , Golden, J. A. , Simmons, R. A. , & Grinspan, J. B. (2012). Delayed myelination in an intrauterine growth retardation model is mediated by oxidative stress upregulating bone morphogenetic protein 4. Journal of Neuropathology & Experimental Neurology, 71, 640–653. 22710965 10.1097/NEN.0b013e31825cfa81 PMC3390978

[glia23216-bib-0059] Resch, B. , Neubauer, K. , Hofer, N. , Resch, E. , Maurer, U. , Haas, J. , & Muller, W. (2012). Episodes of hypocarbia and early‐onset sepsis are risk factors for cystic periventricular leukomalacia in the preterm infant. Early Human Development, 88, 27–31. 21752559 10.1016/j.earlhumdev.2011.06.011

[glia23216-bib-0060] Rideau Batista Novais, A. , Pham, H. , Van de Looij, Y. , Bernal, M. , Mairesse, J. , Zana‐Taieb, E. , … Baud, O. (2016). Transcriptomic regulations in oligodendroglial and microglial cells related to brain damage following fetal growth restriction. Glia, 64, 2306–2320. 27687291 10.1002/glia.23079

[glia23216-bib-0061] Robinson, S. , Li, Q. , Dechant, A. , & Cohen, M. L. (2006). Neonatal loss of gamma‐aminobutyric acid pathway expression after human perinatal brain injury. Journal of Neurosurgery, 104, 396–408. 16776375 10.3171/ped.2006.104.6.396 PMC1762128

[glia23216-bib-0062] Rouse, D. J. , Hirtz, D. G. , Thom, E. , Varner, M. W. , Spong, C. Y. , Mercer, B. M. , … Roberts, J. M. Eunice Kennedy Shriver NICHD Maternal‐Fetal Medicine Units Network (2008). A randomized, controlled trial of magnesium sulfate for the prevention of cerebral palsy. The New England Journal of Medicine, 359, 895–905. 18753646 10.1056/NEJMoa0801187 PMC2803083

[glia23216-bib-0063] Rousset, C. I. , Chalon, S. , Cantagrel, S. , Bodard, S. , Andres, C. , Gressens, P. , & Saliba, E. (2006). Maternal exposure to LPS induces hypomyelination in the internal capsule and programmed cell death in the deep gray matter in newborn rats. Pediatric Research, 59, 428–433. 16492984 10.1203/01.pdr.0000199905.08848.55

[glia23216-bib-0064] Salmaso, N. , Jablonska, B. , Scafidi, J. , Vaccarino, F. M. , & Gallo, V. (2014). Neurobiology of premature brain injury. Nature Neuroscience, 17, 341–346. 24569830 10.1038/nn.3604 PMC4106480

[glia23216-bib-0065] Scafidi, J. , Hammond, T. R. , Scafidi, S. , Ritter, J. , Jablonska, B. , Roncal, M. , … Gallo, V. (2014). Intranasal epidermal growth factor treatment rescues neonatal brain injury. Nature, 506, 230–234. 24390343 10.1038/nature12880 PMC4106485

[glia23216-bib-0066] Semple, B. D. , Blomgren, K. , Gimlin, K. , Ferriero, D. M. , & Noble‐Haeusslein, L. J. (2013). Brain development in rodents and humans: Identifying benchmarks of maturation and vulnerability to injury across species. Progress in Neurobiology, 106–107, 1–16. 10.1016/j.pneurobio.2013.04.001 PMC3737272 23583307

[glia23216-bib-0067] Shah, D. K. , Doyle, L. W. , Anderson, P. J. , Bear, M. , Daley, A. J. , Hunt, R. W. , & Inder, T. E. (2008). Adverse neurodevelopment in preterm infants with postnatal sepsis or necrotizing enterocolitis is mediated by white matter abnormalities on magnetic resonance imaging at term. Journal of Pediatrics, 153, 170–175. 175 e171. 18534228 10.1016/j.jpeds.2008.02.033

[glia23216-bib-0068] Shankaran, S. , Langer, J. C. , Kazzi, S. N. , Laptook, A. R. , & Walsh, M. (2006). Cumulative index of exposure to hypocarbia and hyperoxia as risk factors for periventricular leukomalacia in low birth weight infants. Pediatrics, 118, 1654–1659. 17015558 10.1542/peds.2005-2463

[glia23216-bib-0069] Silbereis, J. C. , Huang, E. J. , Back, S. A. , & Rowitch, D. H. (2010). Towards improved animal models of neonatal white matter injury associated with cerebral palsy. Disease Models & Mechnisms, 3, 678–688. 10.1242/dmm.002915 PMC2965396 21030421

[glia23216-bib-0070] Silberstein, F. C. , De Simone, R. , Levi, G. , & Aloisi, F. (1996). Cytokine‐regulated expression of platelet‐derived growth factor gene and protein in cultured human astrocytes. Journal of Neurochemistry, 66, 1409–1417. 8627292 10.1046/j.1471-4159.1996.66041409.x

[glia23216-bib-0071] Stephens, B. E. , Bann, C. M. , Watson, V. E. , Sheinkopf, S. J. , Peralta‐Carcelen, M. , Bodnar, A. , … Vohr, B. R. (2012). Screening for autism spectrum disorders in extremely preterm infants. The Journal of Developmental and Behavioral Pediatrics, 33, 535–541. 22926660 10.1097/DBP.0b013e31825fd0af PMC3434239

[glia23216-bib-0072] Tay, T. L. , Mai, D. , Dautzenberg, J. , Fernández‐Klett, F. , Lin, G. , Sagar Datta, M. , … Prinz, M. (2017). Nature Neuroscience, 20, 793–803. 28414331 10.1038/nn.4547

[glia23216-bib-0073] Thompson, D. K. , Chen, J. , Beare, R. , Adamson, C. L. , Ellis, R. , Ahmadzai, Z. M. , … Anderson, P. J. (2016). Structural connectivity relates to perinatal factors and functional impairment at 7years in children born very preterm. Neuroimage, 134, 328–337. 27046108 10.1016/j.neuroimage.2016.03.070 PMC4912891

[glia23216-bib-0074] van Tilborg, E. , Heijnen, C. J. , Benders, M. J. , van Bel, F. , Fleiss, B. , Gressens, P. , & Nijboer, C. H. (2016). Impaired oligodendrocyte maturation in preterm infants: Potential therapeutic targets. Progress in Neurobiology, 136, 28–49. 26655283 10.1016/j.pneurobio.2015.11.002

[glia23216-bib-0075] Travis, K. E. , Adams, J. N. , Ben‐Shachar, M. , & Feldman, H. M. (2015). Decreased and increased anisotropy along major cerebral white matter tracts in preterm children and adolescents. PLoS One, 10, e0142860. 26560745 10.1371/journal.pone.0142860 PMC4641645

[glia23216-bib-0076] Treyvaud, K. , Ure, A. , Doyle, L. W. , Lee, K. J. , Rogers, C. E. , Kidokoro, H. , … Anderson, P. J. (2013). Psychiatric outcomes at age seven for very preterm children: rates and predictors. Journal of Child Psychology and Psychiatry, 54, 772–779. 23347471 10.1111/jcpp.12040 PMC3821531

[glia23216-bib-0077] Tsuji, M. , Saul, J. P. , du Plessis, A. , Eichenwald, E. , Sobh, J. , Crocker, R. , & Volpe, J. J. (2000). Cerebral intravascular oxygenation correlates with mean arterial pressure in critically ill premature infants. Pediatrics, 106, 625–632. 11015501 10.1542/peds.106.4.625

[glia23216-bib-0078] Umekawa, T. , Oman, A. M. , Han, W. , Ikeda, T. , & Blomgren, K. (2015). Resident microglia, rather than blood‐derived macrophages, contribute to the earlier and more pronounced inflammatory reaction in the immature compared with the adult hippocampus after hypoxia‐ischemia. Glia, 63, 2220–2230. 26179283 10.1002/glia.22887 PMC5034822

[glia23216-bib-0079] Ure, A. M. , Treyvaud, K. , Thompson, D. K. , Pascoe, L. , Roberts, G. , Lee, K. J. , … Anderson, P. J. (2016). Neonatal brain abnormalities associated with autism spectrum disorder in children born very preterm. Autism Research, 9, 543–552. 26442616 10.1002/aur.1558

[glia23216-bib-0080] Van Steenwinckel, J. , Schang, A. L. , Sigaut, S. , Chhor, V. , Degos, V. , Hagberg, H. , … Gressens, P. (2014). Brain damage of the preterm infant: new insights into the role of inflammation. Biochemical Society Transactions, 42, 557–563. 24646278 10.1042/BST20130284

[glia23216-bib-0081] Verney, C. , Pogledic, I. , Biran, V. , Adle‐Biassette, H. , Fallet‐Bianco, C. , & Gressens, P. (2012). Microglial reaction in axonal crossroads is a hallmark of noncystic periventricular white matter injury in very preterm infants. Journal of Neuropathology & Experimental Neurology, 71, 251–264. 22318128 10.1097/NEN.0b013e3182496429

[glia23216-bib-0082] Vogan, V. M. , Morgan, B. R. , Leung, R. C. , Anagnostou, E. , Doyle‐Thomas, K. , & Taylor, M. J. (2016). Widespread white matter differences in children and adolescents with autism spectrum disorder. Journal of Autism and Developmental Disorders, 46, 2138–2147. 26899725 10.1007/s10803-016-2744-2

[glia23216-bib-0083] Volpe, J. J. , Kinney, H. C. , Jensen, F. E. , & Rosenberg, P. A. (2011). The developing oligodendrocyte: key cellular target in brain injury in the premature infant. International Journal of Developmental Neuroscience, 29, 423–440. 21382469 10.1016/j.ijdevneu.2011.02.012 PMC3099053

[glia23216-bib-0084] Wang, Y. , Cheng, X. , He, Q. , Zheng, Y. , Kim, D. H. , Whittemore, S. R. , & Cao, Q. L. (2011). Astrocytes from the contused spinal cord inhibit oligodendrocyte differentiation of adult oligodendrocyte precursor cells by increasing the expression of bone morphogenetic proteins. Journal of Neuroscience, 31, 6053–6058. 21508230 10.1523/JNEUROSCI.5524-09.2011 PMC3081104

[glia23216-bib-0085] Zanier, E. R. , Fumagalli, S. , Perego, C. , Pischiutta, F. , & De Simoni, M. G. (2015). Shape descriptors of the “never resting” microglia in three different acute brain injury models in mice. Intensive Care Medicine Experimental, 3, 39. 26215806 10.1186/s40635-015-0039-0 PMC4513020

[glia23216-bib-0086] Zonouzi, M. , Scafidi, J. , Li, P. , McEllin, B. , Edwards, J. , Dupree, J. L. , … Gallo, V. (2015). GABAergic regulation of cerebellar NG2 cell development is altered in perinatal white matter injury. Nature Neuroscience, 18, 674–682. 25821912 10.1038/nn.3990 PMC4459267

[glia23216-bib-0087] Zschocke, J. , Bayatti, N. , Clement, A. M. , Witan, H. , Figiel, M. , Engele, J. , & Behl, C. (2005). Differential promotion of glutamate transporter expression and function by glucocorticoids in astrocytes from various brain regions. Journal of Biological Chemistry, 280, 34924–34932. 16079146 10.1074/jbc.M502581200

