# Supplementary material for: Combined fetal inflammation and postnatal hypoxia causes myelin deficits and autism‐like behavior in a rat model of diffuse white matter injury
Source: Glia. 2017 Sep 19;66(1):78–93. doi: 10.1002/glia.23216 (PMC5724703; doi:10.1002/glia.23216)
Supplement: Supplementary file 4 — Supporting Information Figure 4. [file GLIA-66-78-s004.doc]

**Combined fetal inflammation and postnatal hypoxia causes myelin deficits and autism-like behavior in a rat model of diffuse white matter injury**

Erik van Tilborg^1^, E.J. Marijke Achterberg^2^, Caren M. van Kammen^1^, Annette van der Toorn^3^, Floris Groenendaal^4^, Rick M. Dijkhuizen^3^, Cobi J. Heijnen^5^, Louk J.M.J. Vanderschuren^2^, Manon N.J.L. Benders^4^, Cora H.A. Nijboer^1^

1. Laboratory of Neuroimmunology and Developmental Origins of Disease, University Medical Center Utrecht, Lundlaan 6, 3584EA, Utrecht, The Netherlands.

2. Department of Animals in Science and Society, Division of Behavioural Neuroscience, Faculty of Veterinary Medicine, Utrecht University, Yalelaan 2, 3584CM, Utrecht, The Netherlands.

3. Biomedical MR Imaging and Spectroscopy Group, Center for Image Sciences, University Medical Center Utrecht, Bolognalaan 50, 3584 CJ, The Netherlands.

4. Department of Neonatology, University Medical Center Utrecht, Lundlaan 6, 3584EA, Utrecht, The Netherlands.

5. Laboratory of Neuroimmunology, Department of Symptom Research, University of Texas MD Anderson Cancer Center, 1515 Holcombe Blvd, Unit 384, Houston, TX 77030, USA.

FigS4.eps

7.052 kB

EPS

Supporting Information Figure 4
